# Effects of a prebiotic fibre-enriched bread in older adults: a randomised, placebo-controlled, parallel-groups feasibility study (BiomeBakery)

**DOI:** 10.1016/j.jnha.2026.100939

**Published:** 2026-08-13

**Authors:** Ying Lee, Rebecca L. Colombage, Caitlin V. Hall, Daniel J. Lamport, Claire M. Williams, Piril Hepsomali

**Affiliations:** a School of Psychology and Clinical Language Sciences, University of Reading, Reading, United Kingdom; b Myota Limited, London, United Kingdom

**Keywords:** Prebiotics, Cognition, Depression, Anxiety, Mood, Inflammation

## Abstract

**Objectives:**

To examine the feasibility and effects of consuming an additional 10 g/day of prebiotic fibre blend in a bread roll on cognitive, mood, gastrointestinal outcomes, and peripheral biomarkers in older adults without dementia and with low habitual dietary fibre intake.

**Design:**

A single-centre, randomised, double-blind, placebo-controlled parallel groups design (Registration at clinicaltrials.gov; NCT06796712).

**Setting and participants:**

Fifty-three community-dwelling adults aged 60–80 years without dementia and with low habitual dietary fibre intake.

**Intervention:**

Participants were assigned to either a treatment (fibre-enriched white bread roll) group (n = 28) or a placebo (matched white bread roll) group (n = 25) and consumed their assigned products for 12 weeks.

**Measurements:**

Cognitive, mood, gastrointestinal outcomes, as well as C-reactive protein, lipids, and glucose levels were assessed at the baseline and after 12-weeks.

**Results:**

Placebo consumption for 12 weeks was associated with improvements in memory compared with treatment. However, the treatment group showed a decrease in hostility after 12 weeks, whereas the placebo group showed an increase. The placebo group also demonstrated an increase in HDL cholesterol, while the treatment group showed a decrease. No significant differences were observed for the other outcomes.

**Conclusion:**

Fibre-enriched bread may have selective effects on aspects of mood (e.g., hostility) in ageing populations, although most mood, cognitive, and biological outcomes were unchanged. The observed improvement on mood occurred independently of inflammatory or cardiovascular changes, suggesting potential involvement of the gut–brain axis and the need for additional mechanistic endpoints to clarify the underlying mechanistic pathways. Cognitive and inflammatory changes may require longer interventions, different dosing or populations, highlighting the need for further investigation in larger and more diverse samples.

## 1 Introduction

The global population is ageing rapidly, with the World Health Organization projecting that 22% will be over the age of 60 by 2050 [1]. Ageing brings significant challenges in maintaining quality of life, as it is the leading risk factor for a range of chronic and neurodegenerative conditions [2]. Currently, more than 55 million people are living with dementia worldwide, with this number projected to rise to 152 million by 2050 [3]. This poses a significant social and economic burden with annual costs estimated at US $1 trillion, which is set to double in the next five years [4]. Nearly half of dementia cases are linked to modifiable risk factors such as physical inactivity, midlife obesity, and poor cardiovascular health, with diet also playing a key role [5,6]. Given the limited success of pharmacological interventions [7], nutritional interventions may offer a promising path to prevention and risk reduction [8,9].

Growing recognition of the gut microbiome’s role in supporting brain health through the microbiota–gut–brain axis [10,11] has increased interest in the use of prebiotics as potential interventions to influence human brain and behaviour. Prebiotics, defined by the International Scientific Association for Probiotics and Prebiotics as “*substrate[s] that [are] selectively* utilized *by host microorganisms conferring a health benefit*” [12], occur naturally in foods such as fruits, vegetables, and legumes, and are also added to perishable goods including cereals, biscuits, yogurts, and even beverages. Prebiotics primarily influence brain function and behaviour through the production of short-chain fatty acids (SCFAs) generated when gut bacteria ferment these fibres [13]. SCFAs can affect the brain both directly or indirectly impact the brain via vagal, humoral, endocrine, and immune pathways [13,14]. In particular, SCFAs help regulate systemic chronic inflammation through modulation of immune function and intestinal barrier integrity and may contribute to blood pressure regulation through activation of G-protein-coupled receptors expressed on vascular endothelial and smooth muscle cells [[13], [14], [15], [16]]. SCFAs have also been implicated in the regulation of neurotransmitter systems via gut–brain axis signalling by promoting the release of neurotransmitters such as serotonin (5-HT) and γ-aminobutyric acid (GABA), which may influence brain function through circulating and vagal pathways [17]. In addition to SCFA-mediated effects, certain prebiotic and soluble fibres may influence cardiometabolic health by increasing intestinal viscosity, thereby reducing bile acid reabsorption, stimulating hepatic *de novo* bile acid synthesis from cholesterol, and ultimately lowering circulating cholesterol concentrations [15]. These physiological processes are known to influence cognitive and affective functions [[18], [19], [20]], as well as age related cognitive-decline and neurodegeneration [[21], [22], [23], [24]].

To harness the potential benefits of SCFAs, prebiotics have been investigated for their effects on cognitive and affective processes [[25], [26], [27], [28]]. For example, recent evidence from our group has shown that 10 g/day prebiotic fibre supplementation improved self-reported measures of anxiety, stress, and depression, as well as high-sensitivity C-reactive protein (hs-CRP; a biomarker of inflammation) in individuals with metabolic syndrome following a 12-week intervention [29]. However, current syntheses of human clinical trials assessing the cognitive and affective effects of prebiotics have reported limited efficacy that could be attributable to methodological heterogeneity, including differences in intervention types, dosages, and outcome measures, as well as small sample sizes [30,31]. Furthermore, currently, there is a scarcity of well-designed human clinical trials that assess relevant established and emerging biomarkers and potential mechanisms of action (including but not limited to inflammatory and other blood-based biomarkers, gut microbiome and metabolome markers, and neuroimaging markers [32]), particularly those focusing on community-dwelling older adults exposed to acute, periodic, or chronic cognition-taxing challenges/conditions (e.g., unhealthy diet, sedentary behaviour, age-related decline) [33].

Despite the promising effects of prebiotics and prebiotic fibre on brain and behaviour, adults in the United Kingdom often consume only around 20 g of dietary fibre per day, falling short of the recommended 30 g [34] and creating a “fibre gap” [15,35], which may, in turn, limit their therapeutic potential. Building on the fibre gap and abovementioned methodological gaps and challenges, the current single-centre, 12-week, randomised, double-blind, placebo-controlled parallel groups study, primarily investigated the effects of an additional 10 g daily prebiotic fibre intake (delivered via a bread roll) on cognitive function in healthy older adults with or without subjective cognitive complaints who habitually consume less than ∼20 g of dietary fibre per day. Mood, gastrointestinal symptoms, and peripheral biomarkers (systemic inflammation, lipid profiles, and glucose levels) were included as secondary and exploratory outcomes to assess broader affective and mechanistic pathways.

## 2 Methods

### 2.1 Participants

Participants recruited through existing participant databases within the University of Reading where participants had previously consented to be contacted about research studies, community-based advertising (e.g., posters, leaflets, talks), and Join Dementia Research (https://www.joindementiaresearch.nihr.ac.uk/). Following a telephone pre-screening, participants were invited to take part in the study if they were aged between 60 and 80 years, had normal or corrected-to-normal vision and hearing and a body mass index (BMI) within the range of 18.5 to 29.9 kg/m^2^. Individuals were excluded if they had a severe cognitive impairment, defined as a score of 6 or less on the Telephone Interview for Cognitive Status (TICS; [36,37]), were current smokers, or had any food allergies. Additional exclusion criteria included a high dietary fibre intake (defined as more than 22 g per day on the Fiber Screen; a 18-item tool that assesses the frequency and quantity of consumption of major fibre-rich food groups over the previous week, with responses converted into estimated daily fibre intake [g/day]) [38], and any wheat or gluten intolerance or diagnosis of coeliac disease, made major changes to dietary intake in the past month, or had any diagnosed psychiatric or neurological disorders, including eating disorders. Participants were also excluded if they had uncontrolled cardiometabolic diseases, uncontrolled hypertension, thrombosis-related disorders, thyroid disease, or anaemia. The use of anticoagulants, antiplatelet medications, antidepressants, proton-pump inhibitors, fibre, prebiotic or probiotic supplements, or continuous antibiotic use for more than three days within one month prior to enrolment also led to exclusion. Likewise, individuals who had used weight-loss medication for more than one month prior to screening were not eligible. Further exclusion criteria included any significant gastrointestinal condition affecting nutrient absorption including (but not limited to) inflammatory bowel disease; weight loss surgery; irritable bowel disease; end stage renal disease; active cancer, or treatment for any cancer, in the last three years.

### 2.2 Study design

A single-centre, 12-week, randomised, double-blind, placebo-controlled parallel groups study design protocol was performed between May 2025 and February 2026. The protocol adhered to the Helsinki Declaration and received an approval from the University of Reading Ethics Committee (Reference: UREC 24–36) and was registered at clinicaltrials.gov (NCT06796712). This study was designed and reported in accordance with the CONSORT 2025 statement (see Supplementary Table S1 for the CONSORT checklist) [39].

**Fig. 1:**
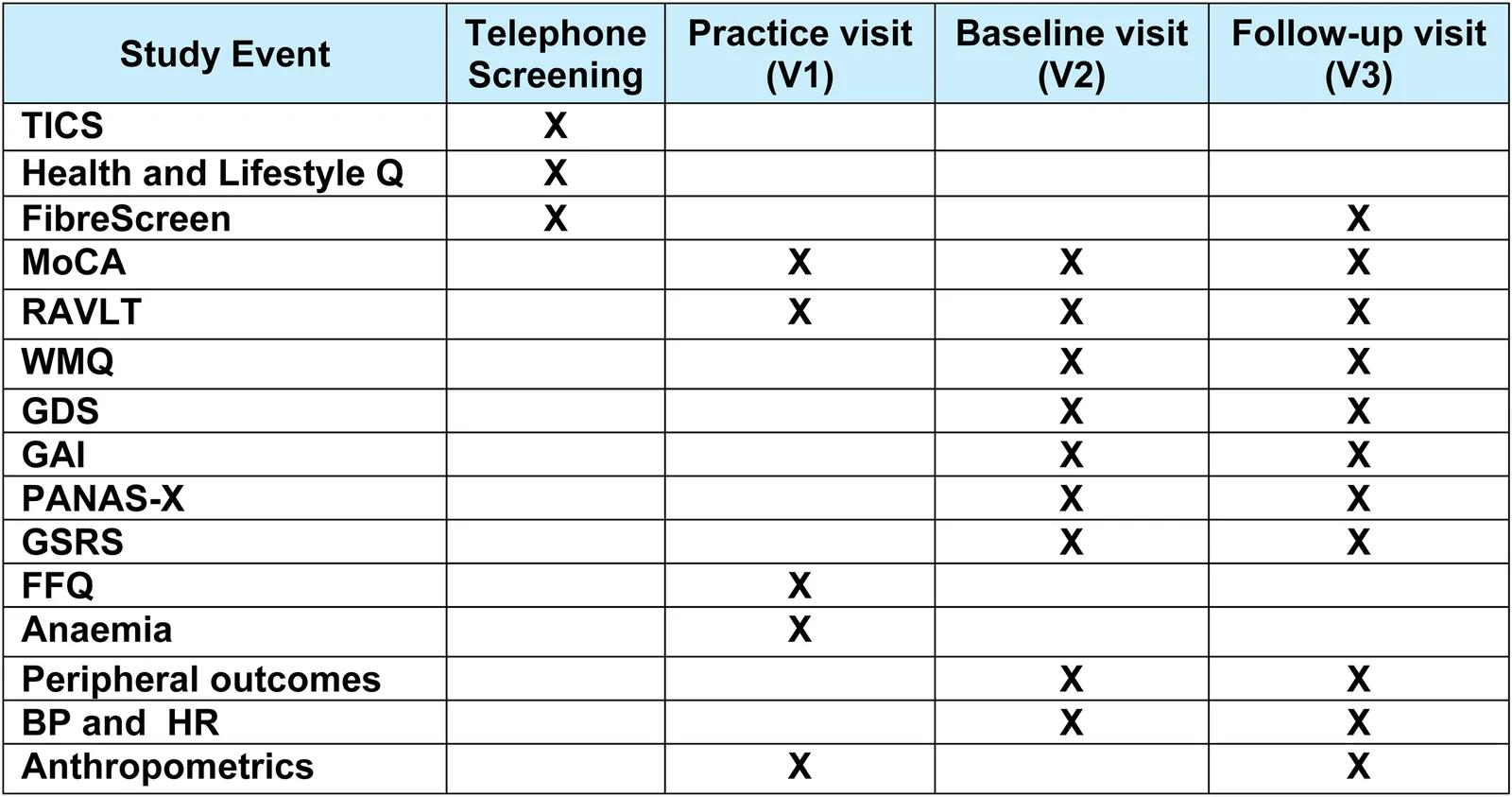
Overview of study schedule.

An overview of the study design is shown in Fig. 1. Participants were randomised (with no stratification factors applied using www.randomizer.org) into a prebiotic fibre-fortified functional bakery product group (containing additional 10 g prebiotic fibre) or a regular bakery product group (containing no additional prebiotic fibre). Participants visited the University of Reading on three occasions: a practice visit (V1), a pre-intervention baseline visit (V2) scheduled approximately 1 week after V1, and a follow-up visit at the end of the intervention (V3). The practice visit (V1) involved obtaining informed consent, followed by a finger-prick (using capillary blood sample volume of 10 μL) assessment for anaemia (using HemoCue Analyzer), and anthropometric measurements including height, weight, waist and hip circumference. To familiarise participants with the experimental procedure, all participants practised cognitive tasks. Participants were also asked to complete the EPIC Norfolk Food Frequency Questionnaire (FFQ) [40] to account for their background diet.

Any participants who tested positive for anaemia (blood haemoglobin levels *<120 g/l for females or <130 g/l for males*) were asked to seek a clinical review and were then reinvited to the study if they were tested negative clinically. The pre-intervention baseline visit (V2) involved a fasted (>8 h) morning finger-prick (capillary) blood sampling, followed by measurement of blood pressure and heart rate, as well as the administration of cognitive, affective, gastrointestinal measures. The follow-up visit at the end of the intervention (V3) was scheduled approximately 12 weeks following the pre-intervention baseline visit (V2) and was identical in procedures to the pre-intervention baseline visit (V2). In addition, fibre screen scores, blood pressure, heart rate, and anthropometric data were also collected at V3.

### 2.3 Treatments

The treatment product consisted of a fibre-enriched white bread roll (68 g bread roll, enriched with 10.9 g of fibre blend per portion [wheat fibre, oat fibre, fructo-oligosaccharides, galacto-oligosaccharides, inulin, resistant maltodextrin, resistant dextrin, partially hydrolysed guar gum, guar fibre] see Supplementary Table S2 for Nutritional Composition). A taste-, appearance-, energy-, and macronutrient-matched white bread roll (68 g bread roll) was used as a placebo (see Supplementary Materials for more information). Both products were manufactured, processed, baked, frozen, packaged, and shipped under identical conditions by Puratos Limited UK, which also held the envelopes containing the treatment allocations. The experimenters did not have access to the random allocation sequence.

Freshly baked treatment and the placebo products were shipped to participants weekly over the course of the intervention period. Participants were instructed to consume one bread roll per day at their preferred times with their preferred food (e.g., as a side for soup, toasting). They were asked to report their time (hh:mm) and context (e.g., half a roll as a side with soup, full roll as a sandwich) of bread roll intake daily to measure compliance. Apart from study products, participants were asked not to modify their dietary intake in any further way.

### 2.4 Outcome measures

All outcome measures were acquired and stored on REDCap unless stated otherwise.

#### 2.4.1 Cognitive, Affective, and Gastrointestinal Outcomes

The Montreal Cognitive Assessment (MoCA), Rey’s Auditory Verbal Learning Task (RAVLT) and Working Memory Questionnaire (WMQ) were used to assess cognitive outcomes.

MoCA [41], a 1-page, 10-min, 30-point pen-and-paper test, assessed memory, visuospatial ability, executive functions, attention, concentration, working memory, language, and orientation to time and place, as well as overall cognitive functioning. The maximum score is 30, with lower scores indicating greater levels of cognitive impairment. MoCA subscores comprised of Memory, Language, Attention, Executive, Visuospatial and Orientation scores [42]. For more information, please see the Supplementary Materials.

A modified RAVLT [43] is a brief (10−15 min) measure of short-term auditory-verbal episodic memory. For more information, please see the Supplementary Materials. Accuracy on the immediate free recall and delayed free recall were used. Different versions of word lists were used across the test sessions, order of word lists used was counterbalanced across participants.

WMQ, a 30-item questionnaire, assessed short-term storage, attention, and executive control components of working memory [44]. Each question was rated on a five-point Likert-type scale, ranging from 0 (“no problem at all”) to 4 (“very severe problem in everyday life”). Three sub-scores were calculated, one for each of the three domains (maximum score of 40 per domain), along with a total score out of 120. Higher scores indicate greater levels of difficulty or more frequent complaints.

Geriatric Depression Scale (GDS), Geriatric Anxiety Inventory (GAI), and Positive and Negative Affect Schedule Expanded (PANAS-X) were used to assess affective outcomes. GDS assesses depressive mood state over the past week. Participants responded “yes” or “no” to 15 items and higher total scores denote higher depressive mood state [45]. GAI assesses anxious mood state over the past week. Participants responded “agree” or “disagree” to 20 items and higher total scores denote higher anxious mood state [46]. The PANAS-X assesses overall positive and negative affect, as well as specific mood factors (Fear, Hostility, Guilt, Sadness, Joviality, Self-Assurance, Attentiveness, Shyness, Fatigue, Serenity, and Surprise) over the past few weeks, using 60 adjectives (30 positive and 30 negative). Participants rate each item on a 5-point Likert scale ranging from 1 (“not at all”) to 5 (“extremely”). Higher scores indicate greater intensity of the corresponding affect or mood state [47].

Gastrointestinal Symptom Rating Scale (GSRS) [48] was used to assess overall and specific gastrointestinal symptoms, including reflux, abdominal pain, indigestion, diarrhoea and constipation over the past week. Participants responded to 15 items on a 7-point Likert scale (“no discomfort at all” to “very severe discomfort”), where higher scores represent worse gastrointestinal symptoms.

#### 2.4.2 Peripheral outcomes

High sensitivity C-Reactive protein levels (hs-CRP; mg/L), lipid profiles (total-cholesterol [mmol/l], Low density lipoprotein (LDL) - cholesterol [mmol/l], High Density lipoprotein (HDL) - cholesterol [mmol/l], triglycerides [mmol/l]), Haemoglobin A1c (HbA1c) [mmol/l]), were measured by utilising finger-prick (capillary) blood tests (Thriva Limited, UK). Blood samples (maximum 1700 μL in total per participant across all study visits) were collected at the Centre for Integrative Neuroscience and Neurodynamics (CINN), University of Reading after an 8 -h minimum fast and were returned to Thriva Limited UK within 5 days of collection. Once samples arrived at the lab, Serum Separator Tubes were immediately centrifuged to separate the blood serum in order to prevent sample decay. The samples are then processed within a 48 -h window by using a Roche Cobas c503 platform.

#### 2.4.3 Anthropometric, blood pressure, and heart rate outcomes

The anthropometric measures included height (cm) and weight (kg) (to estimate body mass index) as well as waist and hip circumference (cm) (to estimate waist to hip ratio). Waist and hip circumference measures were measured twice and averaged. Systolic and diastolic blood pressure (mmHg) and heart rate (bpm) were measured thrice and averaged.

#### 2.4.4 Demographic and lifestyle factors

Weekly duration of high-intensity exercise, alcohol, coffee and tea intake (assessed with a Health and Lifestyle Questionnaire), years of education as well as energy intake (assessed with EPIC-Norfolk food frequency questionnaire (FFQ); analysed using FETA Version 2.53 [49]) were also collected. The EPIC-Norfolk Food Frequency Questionnaire is a semi-quantitative FFQ designed to assess habitual dietary intake over the preceding 12 months. It comprises 130 food items with response options ranging from ‘never or less than once a month’ to ‘6+ per day’, in addition to an open-ended section allowing participants to report other foods not included in the list.

#### 2.4.5 Safety and tolerability

Safety and tolerability were assessed by monitoring and reporting of adverse events throughout the study. Adverse events were recorded and reported as per Incident reporting in Human Interventional Studies at the University of Reading Safety Note 59 [50]. Blood test results were also reviewed for any signs of infection or acute inflammatory responses, with findings escalated according to a predefined scale of clinical urgency as follows – Medium Escalation: review with a GP within 7 days; High Escalation: review with a GP within 24 h or to call 111 if GP is unreachable; Very High Escalation: notification of participants within 1 h and review with a GP the same day or to call 111 as this level of escalation may signify a pathophysiological state that is life-threatening or of clinical significance to require immediate action.

### 2.5 Statistical analyses

Data were analysed using JASP 0.19.0.0 and R 4.5.2 and R Studio Version: 2026.01.0 + 392. Prior to analysis, data distribution was visualised for outliers. Shapiro-Wilk tests were performed to test for normality of the data within each group, Levene’s tests tested for equality of variance between groups. For all analyses, two-tailed significance level was set at *p* < 0.05. Bonferroni correction was applied to correct for multiple testing (*p^adj^* < 0.05). Unblinding of group randomisation was carried out after all statistical analyses was completed.

The primary endpoint was the MoCA total score and the secondary endpoints were all other cognitive, mood, gastrointestinal outcomes and peripheral biomarker levels. Analyses were conducted on an intention-to-treat basis. For all endpoints, differences in change from baseline scores between groups were assessed using separate (i) two-sample t-tests or Mann–Whitney U tests and (ii) ANCOVAs (adjusted for age, gender, and BMI). For participant characteristics, between-group differences at specific time points were assessed using two-sample t-tests, Mann–Whitney U tests, or Pearson’s chi-squared tests, as appropriate.

## 3 Results

### 3.1 Participants

**Fig. 2:**
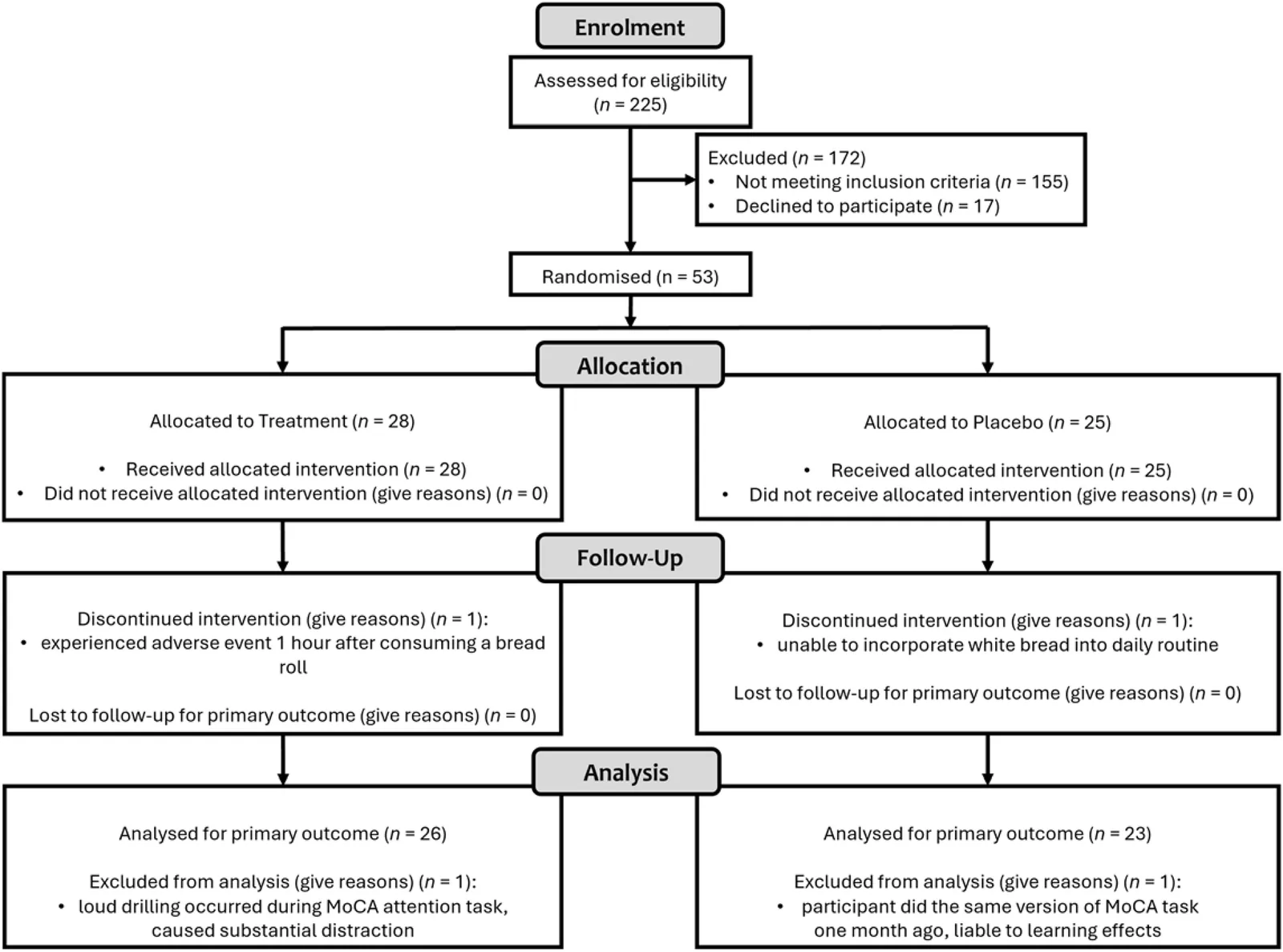
Consolidated Standards of Reporting Trials (CONSORT) flow diagram.

Fig. 2 shows a Consolidated Standards of Reporting Trials (CONSORT) flow diagram [39]. Two hundred and twenty-five participants underwent telephone screening, of which 53 were randomised into Treatment (n = 28) and Placebo (n = 25) group. One participant from each group discontinued the intervention (due to an adverse event and due to difficulty incorporating bread into daily routine), leaving 51 participants who completed the study; two were excluded from analyses due to data quality issues affecting the primary outcome. The remaining 49 participants (Treatment group: n = 26, mean age = 65.5 years, 54% female; Placebo group: n = 23, mean age = 66.6 years, 44% female) were included in the statistical analyses, with no significant differences between the groups in participant characteristics (Table 1), including dietary fibre intake, which remained comparable post-intervention. Sensitivity analysis indicated that the present sample size (n = 53) would allow the detection of moderate-to-large effect sizes with 80% power at an alpha level of 0.05.

**Table 1:** Participant characteristics.

	Treatment group (*n* = 26)		Placebo group (*n* = 23)		Group difference
	Mean	S.D.	Mean	S.D.	Sig. (*p*)
Age (yrs)^2^	65.54	3.86	66.61	6.09	0.89
Body mass index at screening (kg/m^2^)^2^	24.60	1.88	24.82	2.58	0.77
Years of education (yrs)^2^	15.04	2.85	14.20	3.20	0.25
Days between visits 2 and 3 (days)^2^	84.04	0.20	84.43	1.31	0.24
Intervention days missed (days)^2^	2.00	2.45	1.43	2.19	0.45
Intervention percentage frozen (%)^2^	60.57	45.88	62.05	43.47	0.91
Energy intake (kcal/day)^2^	1704.43	582.68	1767.82	554.43	0.54
Fibre intake at screening (g/day)^2^	16.32	4.46	16.42	3.32	0.82
Fibre intake at follow-up (g/day)^2^	18.12	6.14	18.44	5.62	0.96
Alcohol intake (units/week)^2^	9.77	7.77	8.55	8.26	0.46
Tea & coffee intake (cups/week)^1^	27.25	12.95	24.14	15.59	0.45
High intensity exercise (hrs/week)^2^	2.19	1.87	1.60	2.87	**0.05**
TICS score^2^	30.65	3.36	29.00	4.16	0.11
	**Ratio**	**Prop.**	**Ratio**	**Prop.**	**Sig. (*p*)**
Sex (F/M, proportion F)^3^	14/12	0.54	10/13	0.44	0.66
Adverse events (yes/no; proportion yes)^3^	8/18	0.31	9/14	0.39	0.75

1 Independent *t*-test, two-tailed.

2 Mann–Whitney U test, two-tailed.

3 Pearson’s chi squared test, two-tailed.

### 3.2 Cognitive outcomes

There was a significant difference in MoCA memory score (*F*_(1,44)_ = 12.66, *p_adj_*<.001, η_p_^2^ = 0.22), where the placebo group showed a greater improvement in memory after 12-weeks than the treatment group. There were no significant differences in other cognitive measures (all *ps*>0.05) (Table 2).

**Table 2:** Change from baseline scores for each group for cognitive outcomes.

	Treatment group			Placebo group			Group difference	
	N	Mean	S.D.	N	Mean	S.D.	Sig. (*p*^1^)	Sig. (*p_adj_*^2^)
**MoCA total score**	26	0.92	2.06	23	2.00	1.78	0.07	0.11
*MoCA memory*	26	1.04	2.46	23	4.09	3.10	**<.001**	**<.001**
*MoCA language*	26	0.23	0.59	23	0.13	0.87	0.42	0.60
*MoCA attention*	26	0.19	0.69	23	0.09	1.04	0.53	0.73
*MoCA executive*	26	−0.04	0.82	23	0.22	0.85	0.35	0.42
*MoCA visuospatial*	26	0.00	0.57	23	−0.09	0.67	0.76	0.45
*MoCA orientation*	26	−0.04	0.45	23	−0.04	0.64	0.83	0.62
**RAVLT immediate recall accuracy**	26	−0.27	1.78	22	0.27	1.88	0.32	0.42
**RAVLT delayed recall accuracy**	24	0.25	2.21	23	1.52	2.78	0.12	0.11
**WMQ total score**	26	−1.58	5.77	23	−2.35	8.04	0.59	0.67
*WMQ short-term storage*	26	−1.04	2.36	23	−0.91	3.78	0.90	0.85
*WMQ short-term attention*	26	−0.62	2.56	23	−0.96	3.43	0.73	0.68
*WMQ short-term executive control*	26	0.08	2.94	23	−0.48	2.76	0.59	0.42

1 Mann–Whitney U test, two-tailed.

2 ANCOVA test, two-tailed, adjusted for age, BMI and gender.

Additional sensitivity analyses were conducted for the cognitive outcomes that showed significant between-group differences at baseline (MoCA total score, MoCA memory, RAVLT immediate recall, RAVLT delayed recall, and the WMQ storage subscale). These analyses examined change from baseline while adjusting for baseline values, age, sex, and BMI. The findings remained largely unchanged, with no significant intervention effects observed for any cognitive outcome. However, following adjustment, the effect for MoCA memory was attenuated to trend-level significance. Full results are presented in Supplementary Table S6.

### 3.3 Affective and gastrointestinal outcomes

Of the affective outcomes, there was a significant difference in self-reported hostility (*F*_(1,44)_ = 4.23, *p_adj_*<.05, η_p_^2^ = 0.09), where the placebo group showed an increase in hostility after 12-weeks, while the treatment group showed a reduction in hostility. There were no significant differences in other affective nor gastrointestinal measures (all *ps*>0.05) (Table 3).

**Table 3:** Change from baseline scores for each group for affective and gastrointestinal outcomes.

	Treatment group			Placebo group			Group difference	
	N	Mean	S.D.	N	Mean	S.D.	Sig. (*p*^1^)	Sig. (*p_adj_*^2^)
**GDS total**	26	0.12	1.14	23	−0.48	1.41	0.13	0.16
**GAI total**	26	0.04	1.64	23	−0.52	2.15	0.38	0.31
**PANAS-X Negative Affect**	26	0.04	2.66	23	0.17	2.55	0.71	0.82
**PANAS-X Positive Affect**	26	−0.88	6.14	23	0.65	3.56	0.57	0.30
*PANAS-X Fear*	26	0.12	1.56	23	0.48	1.97	0.79	0.46
*PANAS-X Hostility*	26	−0.85	2.41	23	0.30	1.69	**0.04**	**0.05**
*PANAS-X Guilt*	26	−0.46	1.21	23	−0.17	1.47	0.37	0.47
*PANAS-X Sadness*	26	0.38	2.23	23	0.09	1.68	0.62	0.58
*PANAS-X Joviality*	26	−1.19	5.59	23	0.43	3.22	0.36	0.23
*PANAS-X Self-assurance*	26	0.19	4.22	23	0.74	2.91	0.22	0.57
*PANAS-X Attentiveness*	26	−0.46	2.04	23	0.57	1.78	0.22	0.08
*PANAS-X Shyness*	26	−0.04	1.64	23	−0.04	1.55	0.60	0.99
*PANAS-X Fatigue*	26	0.00	3.10	23	0.13	1.74	0.68	0.88
*PANAS-X Serenity*	26	0.19	1.41	23	−0.04	1.26	0.54	0.63
*PANAS-X Surprise*	26	0.46	1.61	23	0.35	1.58	0.61	0.75
**GSRS total score**	26	0.06	0.30	23	0.00	0.24	0.51	0.35
*GSRS Reflux*	26	0.17	0.49	23	0.15	0.51	0.93	0.83
*GSRS Abdominal Pain*	26	−0.04	0.27	23	0.01	0.36	0.99	0.50
*GSRS Indigestion*	26	0.15	0.55	23	−0.04	0.47	0.42	0.17
*GSRS Diarrhoea*	26	−0.04	0.58	23	−0.13	0.45	0.06	0.31
*GSRS Constipation*	26	0.04	0.26	23	0.06	0.80	0.70	0.89

1 Mann–Whitney U test, two-tailed for all except for PANAS-X Serenity which was independent *t*-test, two-tailed.

2 ANCOVA test, two-tailed, adjusted for age, BMI and gender.

### 3.4 Peripheral and anthropometric outcomes

For the peripheral outcomes, there was a significant difference in blood levels of HDL cholesterol (*F*_(1,42)_ = 5.80, *p_adj_*<.05, η_p_^2^ = 0.12), where the placebo group showed an increase in HDL cholesterol after 12-weeks, while the treatment group showed a reduction in in HDL cholesterol. There were no significant differences in all other peripheral and anthropometric measures (all *ps*>0.05) (Table 4).

**Table 4:** Change from baseline scores for each group for peripheral and anthropometric outcomes.

	Treatment group			Placebo group			Group difference	
	N	Mean	S.D.	N	Mean	S.D.	Sig. (*p*^1^)	Sig. (*p_adj_*^2^)
hs-C Reactive Protein [mg/L]	23	−0.37	3.48	19	0.17	1.36	1.00	0.75
Total cholesterol [mmol/l]	26	0.07	0.43	21	0.20	0.94	0.36	0.45
HDL cholesterol [mmol/l]	26	−0.09	0.19	21	0.06	0.22	**0.01**	**0.02**
LDL cholesterol [mmol/l]	26	0.10	0.42	21	0.15	0.77	0.55	0.68
Non-HDL cholesterol [mmol/l]	26	0.15	0.44	21	0.15	0.83	0.88	0.87
Total cholesterol/HDL ratio	26	0.20	0.39	21	0.00	0.54	0.21	0.21
Triglyceride/HDL ratio	26	0.16	0.48	21	−0.04	0.31	0.17	0.15
Triglycerides [mmol/l]	26	0.14	0.48	21	0.00	0.37	0.74	0.32
HbA1c [mmol/l]	23	−0.35	1.99	19	−0.42	2.99	0.89	0.93
Body mass index [kg/m^2^]	26	−0.07	0.50	23	−0.17	0.55	0.47	0.53
Waist circumference [cm]	26	−0.25	2.78	23	−0.64	3.40	0.66	0.69
Hip circumference [cm]	26	1.69	3.76	23	0.79	3.64	0.40	0.59
Waist-to-hip ratio	26	−0.02	0.05	23	−0.01	0.04	0.91	0.83
Blood pressure (systolic) [mmHg]	25	1.51	13.11	22	3.62	9.33	0.73	0.53
Blood pressure (diastolic) [mmHg]	25	2.04	6.40	22	0.11	7.73	0.46	0.35
Heart rate [bpm]	26	1.81	6.62	22	0.74	8.21	0.44	0.54

1 Mann–Whitney U test, two-tailed for all except for BMI, waist and hip circumference which were independent *t*-test, two-tailed.

2 ANCOVA test, two-tailed, adjusted for age, BMI and gender.

### 3.5 Exploratory correlations

Exploratory correlations between (i) MoCA memory scores and affective and peripheral outcomes and (ii) PANAS-hostility scores and cognitive and peripheral outcomes revealed non-significant results (all *ps*>0.05).

### 3.6 Adherence and adverse events

Participants showed an adherence rate of about 98% on average. Approximately 35% of the participants reported experiencing at least one adverse event during the 12-week intervention period. Notably, the proportion of participants who reported these did not differ between the two groups (please see Table 1). As such, it is uncertain whether these adverse events were due to the prebiotic intervention and/or consumption of white bread. Adverse events reported included wind (n = 7), bloating (n = 9), mild constipation (n = 3), abdominal pain (n = 1), loose stools/mild diarrhoea (n = 2), more bowel movements (n = 1) and unspecific stomach discomfort (n = 1). These events were as expected in prebiotic-based intervention studies. One participant experienced severe diarrhoea over a 48 -h period after accidentally consuming mouldy bread.

## 4 Discussion

The current study investigated the feasibility and effects of adding 10 g of fibre per day via a bread roll on cognition, mood, gastrointestinal outcomes, and biomarkers including CRP, lipids, and HbA1c. After 12 weeks, the fibre-enriched white bread roll (treatment) reduced negative mood (specifically hostility), whereas the white bread (placebo) improved memory performance and HDL cholesterol in older adults without dementia who had low habitual fibre intake.

The improvement in memory in the placebo group was somewhat unexpected, as our recent 12-week study using the same prebiotic fibre blend in its original powder form demonstrated improvements in verbal memory (immediate recognition) in healthy older adults with low habitual fibre intake, alongside improvement in the abundance of beneficial bacteria including *Bifidobacterium longum* [51]. Furthermore, previous studies utilising other prebiotic fibre interventions reported improved verbal memory in younger and older populations [31,52], however, systematic reviews and meta-analysis reported null findings [30,53]. Although these discrepancies could be explained by heterogeneity across studies, including (but not limited to) differences in population characteristics (e.g., participants with low dietary protein intake, comorbid conditions…etc.), intervention type (e.g., single prebiotics, multiple prebiotics, or multi-intervention studies involving exercise and/or other adjunct dietary treatments), dose, and duration, and cognitive tests employed, logistical challenges experienced in the current study leading to shipping frozen bread rolls (that were prepared under standardised conditions) to participants may have introduced an unanticipated source of variability. This could plausibly have influenced the bioactive stability of the prebiotic fibre blend by altering structural integrity, viscosity, solubility, and potential for fermentation of the blend throughout freezing-thawing or pre-consumption heat treatment [[54], [55], [56]]. In contrast, these processes are known to increase the resistant starch content of wheat flour, which beneficially increases the availability of fermentable substrate [57], potentially relating to the observed memory changes in the placebo group. Although, these effects would be expected to occur across both study products but may not be equivalent, given that the two breads differed slightly in formulation, with the fibre-enriched bread containing added prebiotic fibre in place of part of the standard bread matrix (58 g bread ingredients plus fibre vs. 68 g full bread formulation). However, this interpretation remains speculative, as the physicochemical stability and fermentability of the prebiotic fibre blend were not directly assessed in the current study, highlighting the need for future trials to examine the impact of processing and delivery format on its structural and functional properties.

Additionally, even a one-week dietary intervention comparing traditionally made sourdough whole-grain bread with industrially produced white bread showed marked personalisation in both bread metabolism and the gut microbiome, further highlighting that individual variability can lead to seemingly counterintuitive outcomes [56,58]. Furthermore, although participation in a dietary intervention study may influence dietary behaviours, as evidenced in Table 1, fibre intake did not change throughout the trial and did not differ between groups, suggesting no substantial changes in fibre intake that would readily explain the observed improvement in memory in the placebo group. However, unmeasured dietary factors or nutrients not assessed in the present study may account for these findings.

Consistent with previous studies showing improvements in mood and mental health [29,31,[59], [60], [61]], participants in the treatment group reported reduced hostility (a component of negative affect), but no changes in other mental health and mood outcomes. As previous studies noted above were conducted in different populations (e.g., individuals with low fibre intake, middle-aged adults, individuals with metabolic syndrome…etc.) and used alternative mood and mental health endpoints, the null findings observed here could be attributable to (i) differences in population characteristics and (ii) the limited sensitivity of the GDS and GAI utilised in the current study to detect effects of nutritional intervention in this population [62]. Alternatively, prebiotics could selectively improve high arousal mood outcomes (hostility) compared to negative low arousal mood outcomes (sadness/fear) or more stable characteristics like depression and anxiety, possibly because the hostility subscale may capture subtle day-to-day fluctuations in mood more readily than the fear or sadness subscale (which can be lower at baseline in healthy older adults, limiting detectable change) [63] or through effects on specific biological mechanisms. In terms of mechanisms, it is important to highlight that although inflammation could contribute to mood improvements following prebiotic consumption particularly in populations with baseline systemic inflammation or cardiometabolic risk (through mechanisms including increased production of short-chain fatty acids, improved gut barrier function, and potential downstream reductions in circulating pro-inflammatory cytokines) [13,29], in the current study, affect-related changes occurred independently of inflammatory changes, although this remains a putative rather than demonstrated mechanism in the absence of direct biomarker evidence. This may involve the hypothesised modulation of tryptophan metabolism [64], regulation of the HPA axis [25], production of SCFAs [13,14], and increased microbial diversity [61], all of which can influence mood- and affect-related neurotransmitters such as serotonin, dopamine, and gamma-aminobutyric acid [11]. Specifically, alterations in tryptophan metabolism and serotonergic signalling [65] and dysregulation of the HPA axis [66] have been implicated in hostility and anger-related states, emphasising the need to measure relevant biomarkers in order to better elucidate the mechanisms underlying these potential effects.

Regarding the peripheral and anthropometric outcomes, unlike our previous prebiotic intervention in individuals with metabolic syndrome [29] and a systematic review and meta-analysis of prebiotic randomised controlled trials in overweight and obese adults [67], we did not observe a reduction in CRP levels in the treatment group. However, consistent with the studies mentioned above and our recent 12-week study using the same prebiotic fibre [51], other endpoints remained unchanged in the current study. Individuals with metabolic syndrome, overweight, or obesity are known have higher baseline CRP levels [68,69], providing more room for improvement. Similarly, improvements in cholesterol and blood pressure following prebiotic use have mostly been observed in individuals with compromised health, including hypertension [70] and dyslipidaemia [71], highlighting once again the possibility of involvement of other mechanisms in the current population. Notably, the only marker that changed was HDL cholesterol, with a decrease observed in the treatment group and an increase in the placebo group. It is well known that circulating HDL concentration alone may not fully reflect its biological functionality, whereas characteristics such as particle size, composition, number, and cholesterol efflux capacity may better capture its effects [72]. Furthermore, paradoxical associations between lower HDL cholesterol levels and adverse health outcomes, including all-cause mortality, cancer, and infection, have been reported, and pharmacological interventions intended to improve cardiovascular risk profiles (e.g., statins) have also been shown to reduce HDL cholesterol levels. Hence, findings should be interpreted in caution and are not intended to be interpreted in isolation as evidence of broader cardiometabolic effects [72].

The current study has several strengths including the incorporation of peripheral biomarkers of inflammation and cardiovascular risk with blood draw times to control for circadian fluctuations, a relatively long intervention duration that increases the likelihood of detecting meaningful changes, comprehensive multidimensional outcome assessments, high participant compliance, and the use of alternate task forms to minimise potential learning effects. However, some caveats should be considered when interpreting the findings. Firstly, although the sensitivity analysis indicated that the current sample size was sufficient to detect moderate-to-large effect sizes, the relatively small sample size combined with the large number of outcomes increases the likelihood of both type I error (due to multiple comparisons) and type II error (due to limited statistical power and the conservative nature of multiplicity correction). While Bonferroni adjustment was, this approach may further increase the risk of type II error. Therefore, replication in a larger, fully powered sample would strengthen the reliability of the findings, as this study was designed as a feasibility trial. Secondly, the absence of direct measures of gut microbiota composition and function, or other potential mechanistic pathways including the HPA axis and tryptophan metabolism limits the ability to fully interpret the observed effects. Thirdly, adherence logs indicate that participants generally consumed the rolls alongside healthy foods, although there was a trend-level (*p* = 0.08) difference in consumption timing, with participants in the placebo group typically consuming them earlier in the day (breakfast or lunch) and participants in the treatment group later in the day (lunch or dinner). As food timing can influence metabolic processes such as glucose regulation, gut motility, and inflammatory responses [73], which may affect cognition and mood, stricter dietary control outside the intervention would further ensure group consistency. Finally, participants were physically active, with most reporting levels of vigorous physical activity consistent with current recommendations (≥75 min/week). In addition, baseline values indicated some between-group differences for certain outcomes (see Supplementary Materials), which were addressed using change-from-baseline analyses. These factors may have limited the potential to detect additional benefits of the intervention owing to reduced scope for improvement. However, sensitivity analyses adjusting for baseline values for the cognitive outcomes that differed significantly at baseline yielded largely unchanged findings, with no significant intervention effects observed for any outcome; the only exception was MoCA Memory, for which the effect was attenuated to trend-level significance, suggesting that baseline differences had minimal influence on the overall pattern of results from the change-from-baseline analyses.

In conclusion, the present feasibility study did not find convincing evidence that 12-week consumption of prebiotic-enriched bread improved cognitive, inflammatory, or cardiometabolic outcomes in older adults. However, a selective reduction in hostility was observed, with no broader effects detected across measures of depression, anxiety, general affect, suggesting that this finding should be interpreted cautiously as a specific and isolated signal rather than a general mood benefit. Nevertheless, fortifying bread with prebiotics proved to be a practical and well-tolerated dietary intervention, as reflected by the consistently high adherence rates. Future research is warranted to test prebiotics in different food matrices, under various preparation and storage conditions and in populations where not only inflammation, but also other relevant biological mechanisms are implicated, while ensuring the incorporation of sufficiently sensitive cognitive and mood assessments, and additional relevant biomarkers (such as microbiome profiling or SCFA quantification) to fully evaluate potential benefits.

## CRediT authorship contribution statement

Ying Lee: Data curation, Formal analysis, Investigation, Writing – original draft; Rebecca L Colombage: Investigation, Writing – review & editing; Daniel L Lamport: Supervision, Writing – review & editing; Claire M Williams: Supervision, Writing – review & editing; Caitlin V Hall: Conceptualization, Funding acquisition, Methodology, Writing – review & editing; Piril Hepsomali: Conceptualization, Data curation, Formal analysis, Funding acquisition, Methodology, Project administration, Supervision, Writing – original draft, Writing – review & editing.

## Declaration of Generative AI and AI-assisted technologies in the writing process

During preparation of this work, ChatGPT was used to assist with grammar, spelling, and sentence structure. No analytical or interpretative content was generated by the model. The authors reviewed and edited all outputs and take full responsibility for the final content.

## Funding

This research was jointly funded by Innovate UK and the Biotechnology and Biological Sciences Research Council (BBSRC) [Grant number = 10123179].

## Data availability statement

The data that support the findings will be available in the University of Reading Research Data Archive at https://researchdata.reading.ac.uk/ following an embargo from the date of publication to allow for commercialisation of research findings.

## Declaration of competing interest

C. V. H. is an employee/shareholder of Myota Limited. The remaining authors declare that the research was conducted in the absence of any commercial or financial relationships that could be construed as a potential conflict of interest. The funders had no role in the design of the study; in the collection, analyses, or interpretation of data; or in the decision to publish the results.
