# Supplementary material for: Effects of a prebiotic fibre-enriched bread in older adults: a randomised, placebo-controlled, parallel-groups feasibility study (BiomeBakery)
Source: J Nutr Health Aging. 2026 Aug 13;30(10):100939. doi: 10.1016/j.jnha.2026.100939 (PMC13495385; doi:10.1016/j.jnha.2026.100939)
Supplement: Supplementary file 1 [file mmc1.docx]

**Supplementary Material**

**Supplementary Table 1. CONSORT 2025 checklist**

| **Section / Topic** | **No** | **CONSORT 2025 checklist item description** | **Reported on page no.** |
| --- | --- | --- | --- |
| **Title and abstract** | | |  |
| Title and structured abstract | 1a | Identification as a randomised trial | 1 and 2 |
|  | 1b | Structured summary of the trial design, methods, results, and conclusions | 2 |
| **Open science** | | |  |
| Trial registration | 2 | Name of trial registry, identifying number (with URL) and date of registration | 2 |
| Protocol and statistical analysis plan | 3 | Where the trial protocol and statistical analysis plan can be accessed | 1 |
| Data sharing | 4 | Where and how the individual de-identified participant data (including data dictionary), statistical code and any other materials can be accessed | 1 |
| Funding and conflicts of interest | 5a | Sources of funding and other support (e.g., supply of drugs), and role of funders in the design, conduct, analysis and reporting of the trial | 1 |
|  | 5b | Financial and other conflicts of interest of the manuscript authors | 1 |
| **Introduction** | | |  |
| Background and rationale | 6 | Scientific background and rationale | 3 and 4 |
| Objectives | 7 | Specific objectives related to benefits and harms | 4 |
| **Methods** | | |  |
| Patient and public involvement | 8 | Details of patient or public involvement in the design, conduct and reporting of the trial | NA |
| Trial design | 9 | Description of trial design including type of trial (e.g., parallel group, crossover), allocation ratio, and framework (e.g., superiority, equivalence, non-inferiority, exploratory) | 5 |
| Changes to trial protocol | 10 | Important changes to the trial after it commenced including any outcomes or analyses that were not prespecified, with reason | NA |
| Trial setting | 11 | Settings (e.g., community, hospital) and locations (e.g., countries, sites) where the trial was conducted | 5 |
| Eligibility criteria | 12a | Eligibility criteria for participants | 4 and 5 |
|  | 12b | If applicable, eligibility criteria for sites and for individuals delivering the interventions (e.g., surgeons, physiotherapists) | NA |
| Intervention and comparator | 13 | Intervention and comparator with sufficient details to allow replication. If relevant, where additional materials describing the intervention and comparator (e.g., intervention manual) can be accessed | 6 and 7 |
| Outcomes | 14 | Pre-specified primary and secondary outcomes, including the specific measurement variable (e.g., systolic blood pressure), analysis metric (e.g., change from baseline, final value, time to event), method of aggregation (e.g., median, proportion), and time point for each outcome | 7, 8, and 9 |
| Harms | 15 | How harms were defined and assessed (e.g., systematically, non-systematically) | 8 and 9 |
| Sample size | 16a | How sample size was determined, including all assumptions supporting the sample size calculation | 9 |
|  | 16b | Explanation of any interim analyses and stopping guidelines | NA |
| Randomisation: |  |  |  |
| Sequence generation | 17a | Who generated the random allocation sequence and the method used | 5 |
|  | 17b | Type of randomisation and details of any restriction (e.g., stratification, blocking and block size) | NA |
| Allocation concealment mechanism | 18 | Mechanism used to implement the random allocation sequence (e.g., central computer/telephone; sequentially numbered, opaque, sealed containers), describing any steps to conceal the sequence until interventions were assigned | 6 |
| Implementation | 19 | Whether the personnel who enrolled and those who assigned participants to the interventions had access to the random allocation sequence | 6 |
| Blinding | 20a | Who was blinded after assignment to interventions (e.g., participants, care providers, outcome assessors, data analysts) | 2, 4, 5, and 9 |
|  | 20b | If blinded, how blinding was achieved and description of the similarity of interventions | 6 |
| Statistical methods | 21a | Statistical methods used to compare groups for primary and secondary outcomes, including harms | 9 |
|  | 21b | Definition of who is included in each analysis (e.g., all randomised participants), and in which group | 9, 12, 13, and 14 |
|  | 21c | How missing data were handled in the analysis | 9 and 10 |
|  | 21d | Methods for any additional analyses (e.g., subgroup and sensitivity analyses), distinguishing prespecified from post-hoc | NA |
| **Results** | | |  |
| Participant flow, including flow diagram | 22a | For each group, the numbers of participants who were randomly assigned, received intended intervention, and were analysed for the primary outcome | 10 |
|  | 22b | For each group, losses and exclusions after randomisation, together with reasons | 10 |
| Recruitment | 23a | Dates defining the periods of recruitment and follow-up for outcomes of benefits and harms | 5 |
|  | 23b | If relevant, why the trial ended or was stopped | NA |
| Intervention and comparator delivery | 24a | Intervention and comparator as they were actually administered (e.g., where appropriate, who delivered the intervention/comparator, how participants adhered, whether they were delivered as intended [fidelity]) | 6 and 7 |
|  | 24b | Concomitant care received during the trial for each group | NA |
| Baseline data | 25 | A table showing baseline demographic and clinical characteristics for each group | 11 |
| Numbers analysed,  outcomes and estimation | 26 | For each primary and secondary outcome, by group:   - the number of participants included in the analysis - the number of participants with available data at the outcome time point - result for each group, and the estimated effect size and its precision (such as 95% confidence interval) - for binary outcomes, presentation of both absolute and relative effect size | 11,12,13, and 14 |
| Harms | 27 | All harms or unintended events in each group | 4 |
| Ancillary analyses | 28 | Any other analyses performed, including subgroup and sensitivity analyses, distinguishing pre-specified from post-hoc | NA |
| **Discussion** | | |  |
| Interpretation | 29 | Interpretation consistent with results, balancing benefits and harms, and considering other relevant evidence | 15, 16, and 17 |
| Limitations | 30 | Trial limitations, addressing sources of potential bias, imprecision, generalisability, and, if relevant, multiplicity of analyses | 17 |

**Supplementary Table 2. Nutritional composition of prebiotic fibre supplement**

| **Typical Values** | **Per 100g** | **Per portion (10g)** | **% DV** |
| --- | --- | --- | --- |
| **Energy** | 200 kcal  840 kJ | 20 kcal  84 kJ | - |
| **Fat** | 0.0g | 0.0g | 0% |
| **Carbohydrate**  *Sugar*  *Dietary fibre* | 100g  1.4g  98g | 10g  0.14g  9.8g | 3%  0.4%  35% |
| **Protein** | 0.0g | 0.0g | 0% |
| **Salt** | 0.0g | 0.0g | 0% |

**Ingredients:** Wheat fibre (gluten free) (2.6g), Oat fibre (gluten free) (1.8g), Fructooligosaccharides (1.4g), Galactooligosaccharides (1.38g), Inulin (1.1g), Resistant dextrin (0.47g), Resistant maltodextrin (0.58g), Partially hydrolysed guar gum (0.5g), guar fibre (0.17g)

**Treatment and Placebo Products (Prebiotic-fibre enriched white bread roll and White bread roll)**

Ingredients used for producing the final fibre-enriched white bread roll (68g, 10.9g fibre) included wheat flour (wheat flour, calcium carbonate, iron, nicotinamide, thiamine), water, metabolic mix (including wheat fibre, oat fibre, fructo-oligosaccharides, galacto-oligosaccharides, inulin, soluble corn fibre, partially hydrolyzed guar gum, guar fibre), yeast, sugar, salt, emulsifiers (mono- and diglycerdies of fatty acids (E471), sodium stearoyl-2-lactylate (E481)), soya flour, vegetable oils (palm, rapeseed), inactive dry yeast, flour treatment agent (ascorbic acid (E300)), enzymes. The same ingredients (excluding the metabolic mix) were used for the placebo white bread roll. Bread rolls had ambient shelf life of up to 7 days.

**Outcome Measures**

***Montreal Cognitive Assessment (MoCA)***

MoCA [1], a 1-page, 10-min, 30-point pen-and-paper test, assessed memory, visuospatial ability, executive functions, attention, concentration, working memory, language, and orientation to time and place, as well as overall cognitive functioning. The maximum score is 30, with lower scores indicating greater levels of cognitive impairment. MoCA sub-scores comprised of Memory, Language, Attention, Executive, Visuospatial and Orientation scores [39]. Memory score is calculated by adding the number of words remembered in the 5-word list free delayed recall, category-cued recall, and multiple choice-cued recall multiplied by 3, 2 and 1, respectively, with a score ranging from 0 to 15. Language score is calculated by adding raw scores for animal naming, sentence repetition and letter fluency, with a score ranging from 0 to 6. Attention score is calculated by adding the raw scores for digit span forward and backward, letter A tapping, serial-7 subtraction, sentence repetition, and the words recalled in both immediate recall trials, with a score ranging from 0 to 18. Executive score is calculated by adding raw scores for the modified Trail-Making Test Part B, clock drawing, digit span forward and backward, letter A tapping, serial-7 subtraction, letter fluency, and abstraction, with a score ranging from 0 to 13. Visuospatial score is calculated by adding the raw scores of copy drawing, clock drawing, and animal naming, with a score ranging from 0 to 7. Orientation score is the sum of points for the orientation section of the MoCA, with a score ranging from 0 to 6.

***Rey’s Auditory Verbal Learning Task***

A modified RAVLT [2] is a brief (10-15 minutes) measure of short-term auditory-verbal episodic memory. It involved five sequential presentations of a 15-word list (List A, Trials 1 through 5). Each pre-recorded word is presented through a laptop loudspeaker at a rate of one word per second, the order of word presentation remained fixed across trials. Following each presentation, the participant is asked to recall verbally as many words as possible within one minute. After the fifth recall, a new 15-word list (List B) is presented. The participant is asked to recall as many words as possible from List B within one minute. Immediately following this, the participant is asked to recall as many words as possible from List A (post-interference recall). After approximately 30 minutes, the participant is asked again to recall as many words as possible from List A.

**Supplementary Tables 3 to 5. Baseline and follow-up mean (±SD) values for treatment and placebo groups across all outcomes**

**Supplementary Table 6. Sensitivity analyses examining change from baseline while adjusting for baseline values, age, gender, and BMI for the cognitive outcomes that showed significant between-group differences at baseline**

|  | **Treatment group** | | | **Placebo group** | | | **Sig. (*p_adj_^*^*)** | |
| --- | --- | --- | --- | --- | --- | --- | --- | --- |
|  | **N** | **Mean** | **S.D.** | **N** | **Mean** | **S.D.** |  |  |
| MoCA total score | 26 | 0.92 | 2.06 | 23 | 2.00 | 1.78 | 0.98 |  |
| *MoCA memory* | 26 | 1.04 | 2.46 | 23 | 4.09 | 3.10 | 0.15 |  |
| RAVLT immediate recall accuracy | 26 | -0.27 | 1.78 | 22 | 0.27 | 1.88 | 0.72 |  |
| RAVLT delayed recall accuracy | 24 | 0.25 | 2.12 | 23 | 1.52 | 2.78 | 0.75 |  |
| *WMQ short-term storage* | 26 | -1.04 | 2.46 | 23 | -0.91 | 2.36 | 0.21 |  |
|  |  |  |  |  |  |  |  |  |
| ^*^: ANCOVA test, two-tailed, adjusted for age, BMI, gender, and baseline performance/scores | | | | | | | | |
|  |  |  |  |  |  |  |  |  |
|  |  |  |  |  |  |  |  |  |

**References**

1. Nasreddine, Z.S., et al., *The Montreal Cognitive Assessment, MoCA: a brief screening tool for mild cognitive impairment.* J Am Geriatr Soc, 2005. **53**(4): p. 695–9.
2. Julayanont, P., et al., *Montreal Cognitive Assessment Memory Index Score (MoCA-MIS) as a predictor of conversion from mild cognitive impairment to Alzheimer's disease.* J Am Geriatr Soc, 2014. **62**(4): p. 679–84.
